# Three-Way Translocation t(12;15;17) (p13;q24;q21) Found in Acute Promyelocytic Leukemia with Basophilic Differentiation

**DOI:** 10.3390/hematolrep16020037

**Published:** 2024-06-12

**Authors:** Sara Frazzetto, Lara Gullo, Gabriele Sapuppo, Manlio Fazio, Cristina Lo Faro, Giuliana Giunta, Ignazio Caravotta, Elisa Mauro, Marina Silvia Parisi, Anna Maria Triolo, Nunziatina Laura Parrinello, Maria Letizia Consoli, Loredana També, Daniela Cambria, Sara Marino, Grazia Scuderi, Francesco Di Raimondo

**Affiliations:** 1 Division of Hematology, Azienda Policlinico-San Marco, 95123 Catania, Italy; lara.gullo@virgilio.it (L.G.); g.sap.95@gmail.com (G.S.); manliofazio@hotmail.it (M.F.); cristinalf94@hotmail.it (C.L.F.); giulianagiunta24@gmail.com (G.G.); caravottaignazio@gmail.com (I.C.); elixmauro@hotmail.com (E.M.); marinaparisi@hotmail.it (M.S.P.); cit.triolo@libero.it (A.M.T.); lauraparrinello@tiscali.it (N.L.P.); marzia.consoli@libero.it (M.L.C.); loredana.tambe@policlinico.unict.it (L.T.); cambriad@tiscali.it (D.C.); saramarino92@virgilio.it (S.M.); graziascuderi@hotmail.it (G.S.); francesco.diraimondo@unict.it (F.D.R.); 2 Postgraduate School of Hematology, University of Catania, 95123 Catania, Italy

**Keywords:** acute myeloid leukemia, acute promyelocytic leukemia, basophils, cytogenetic, translocation, treatment

## Abstract

Acute promyelocytic leukemia is a rare form of acute myeloid leukemia in which immature promyelocytes abnormally proliferate in the bone marrow. In most cases, the disease is characterised by the translocation t(15;17) (q24;q21), which causes the formation of PML::RARA, an oncogenic fusion protein responsible for blocking myeloid differentiation and survival advantage. Here, we present a case of acute promyelocytic leukemia with two unusual features: basophilic differentiation and a three-way translocation involving chromosomes 12, 15 and 17. In the few cases reported, basophilic differentiation was associated with a poor prognosis. In contrast, our patient responded promptly to the standard treatment with all-trans-retinoic acid (ATRA) and arsenic trioxide (ATO) and obtained complete remission. To our knowledge, this is the first report of basophilic acute promyelocytic leukemia with the three-way translocation t(12;17;15) (p13; q24;q21).

## 1. Introduction

Acute promyelocytic leukemia (APL) is a rare haematological malignancy that usually presents with abnormal white blood cell counts (WBCc), low platelets (PLT) and consumptive coagulopathy, requiring prompt treatment for the high risk of fatal bleeding [1].

In 95% of cases, APL is characterised by a reciprocal balanced translocation between chromosomes 15 and 17, t(15;17) (q24;q21), which produces a fusion between the PML (promyelocytic leukemia) gene and the retinoic acid receptor (RARA) gene [2]. The understanding of the pathogenetic role of the PML::RARA fusion gene led to a highly effective treatment with all-trans-retinoic acid (ATRA) and arsenic trioxide (ATO). Physiologically, PML transcripts interact with proteins involved in the regulation of several cellular pathways affecting apoptosis, senescence, epigenetic regulation and transcription of haematopoietic stem cells. The RARA protein is a nuclear receptor activated by retinoids, a class of molecules that are vitamin A derivates. RARA acts as a differentiating agent of myeloid haematopoietic cells. In APL, PML::RARA interacts with several classes of protein resulting in the block of myeloid differentiation at the promyelocytic stage [3]. Moreover, PML::RARA confers survival and proliferative advantage to leukemic cells, which leads to the progressive accumulation of promyelocytes in the bone marrow. In rare cases, other cytogenetic abnormalities may occur, leading to the creation of PML::RARA or other fusion proteins that may act as PML::RARA [4,5].

A possible presentation of APL is with a high basophil count. The basophilic trait is found in up to one-third of all APL cases and is probably underdiagnosed by cytomorphology and flow cytometry assays. This feature, together with CD203c/CD22 positivity and older age, seems to predict the risk of severe bleeding either at presentation or after starting therapy [6,7]. With this paper, we aim to describe a peculiar case of APL presenting with basophilia and a three-way translocation involving chromosomes 12, 15 and 17. To our knowledge, this is the first report in the literature on a basophilic APL with such an unusual karyotype.

## 2. Materials and Methods

Bone marrow blood was collected from the patient with her informed consent. The complete and differential blood count was performed using an automated haematology analyser. A total of 15 × 10^6^ cells were incubated at 37 °C and 5% CO_2_, in CHANG medium BMC with gentamicin (Irvine Scientific, Santa Ana, CA, USA) for 24 h. Conventional cytogenetic analysis was performed [8]. Cells were incubated using standard protocols and processed by conventional methods, and chromosomes were stained with Giemsa. G-banding was performed by tripsin treatment stained with Giemsa (GTG-banding technique) at 300 band resolution. A total of 20 metaphases were analysed [9]. The karyotype was described according to the International System for Human Cytogenetic Nomenclature (ISCN) [10].

Flow cytometry analysis was performed by DX-Flex 13-colour cytometer (Beckman Coulter, Brea, CA, USA). Due to *punctio sicca*, bone marrow blood samples obtained were not sufficient to proceed with further analysis such as fluorescence in situ hybridisation (FISH).

PML::RARA was detected with quantitative real-time polymerase chain reaction (q RT-PCR) [11].

## 3. Case Presentation

**Figure 1:**
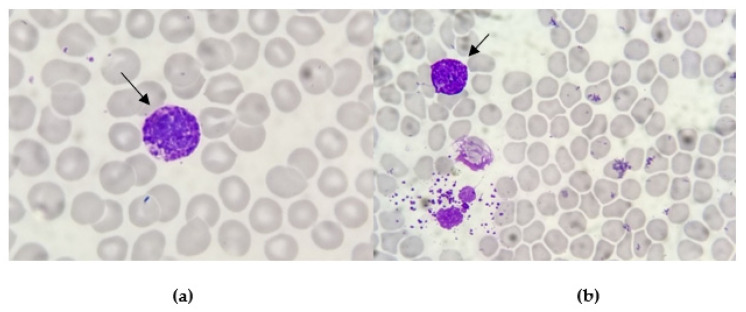
Smear observation of bone marrow aspirates: the cytoplasm of these large cellular elements is characterised by the presence of many basophilic granules (arrow pointed). The line between nucleus and cytoplasm is not well marked. These coarse, dark blu/purple granules totally obscure the nucleus (**a**) or are scattered outside the cells after degranulation (**b**).

A Caucasian 49-year-old woman was admitted to our Hematology Division in January 2023 for severe pancytopenia (Hb 8.1 gr/dL, PLT 64.000/m^3^, WBC 1160/m^3^, ANC 290/m^3^). The patient presented asthenia, fever, back pain, post-prandial emesis and profuse diarrheal discharges. At physical examination, the patient had facial swelling, a palpable lymphadenopathy in the submandibular region with a hard wooden consistency and multiple haematomas in the lower limbs. Computer tomography (CT) confirmed the presence of retromandibular lymphadenopathy. We performed bone marrow (BM) aspirate and bone trephine biopsy. At the smear observation, the bone marrow had a decreased cellularity. Some of the visible cellular elements displayed a basophilic differentiation (Figure 1). 

Flow cytometry analysis showed the presence of 38% of CD33+, CD38+ and CD71+ cells, with the partial expression of CD34 (24%), CD117 (10%) and CD123 (34%). Furthermore, 12% of CD123+ cells expressed HLA-DR, CD38 and CD33 and appeared to be basophils.

**Figure 2:**
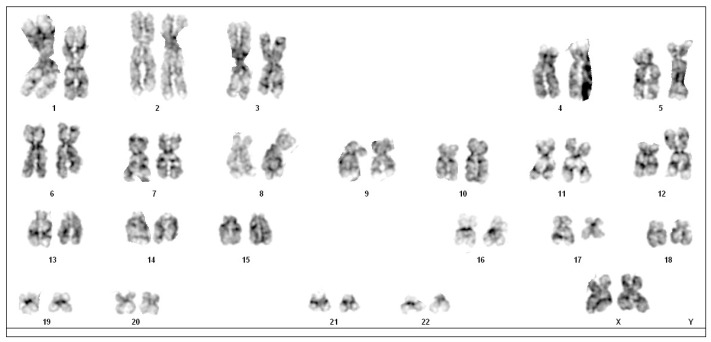
Karyotype of the patient with a translocation t(12;15;17) (p13; q24;q21).

Cytogenetic analysis was performed with the G-banding technique. The 35% of obtained metaphases presented a triple translocation involving chromosomes 12, 15 and 17, namely t(12;15;17), with breakpoints at 12p13, 15q24 and 17q21. No other consistent structural or numerical abnormalities were detected. Thus, the chromosome aberration was interpreted as 46 XX, t(12;15;17) (p13;q24;q21). Due to the exiguity of the bone marrow sample, it was not possible to perform fluorescence in situ hybridisation (FISH) (Figure 2).

FLT3 and NPM1 resulted umutated/as wild type at molecular biology analysis. Lastly, the histopathology essay documented increased bone marrow (BM) cellularity (near 100%), mostly represented by immature cells with lax chromatin and small central nucleoli. Cells immunophenotype on histological samples was MPO+, CD33+, CD43+, CD13+/−, CD34+, CD56−, CD4−, CD103−, TdT−, CD20− and CD3−.

**Figure 3:**
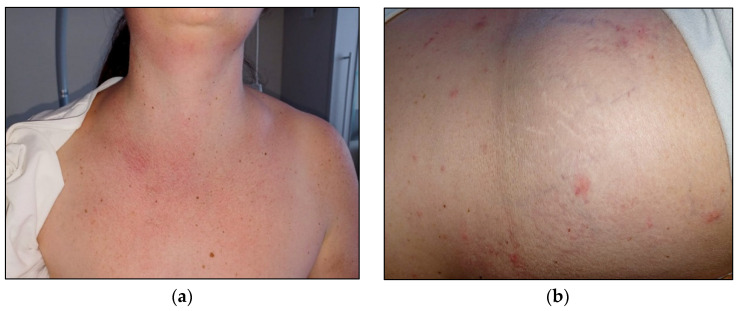
Skin rash (**a**) and urticarial-like skin lesions (**b**).

Interestingly, during the first days of hospitalisation, the patient suffered from recurrent episodes of urticarial-like skin rash, particularly in the chest area and upper limbs, which was associated with severe itching and was responsive to antihistamine treatment (Figure 3).

In this regard, the Flow Cytometry Laboratory required another sample to improve the immunophenotype characterisation. The BM aspirate failed for dry tap, so we decided to opt for peripheral blood (PB) immunophenotyping. The subsequent analysis showed the presence of 38% of cellular elements CD9+, CD11 b+, CD22+, CD25+/−, CD33+, CD43+, CD71+, CD123+ and CD45+, and positivity for the CD34+ immaturity marker showed in only 1.5% of cells.

**Figure 4:**
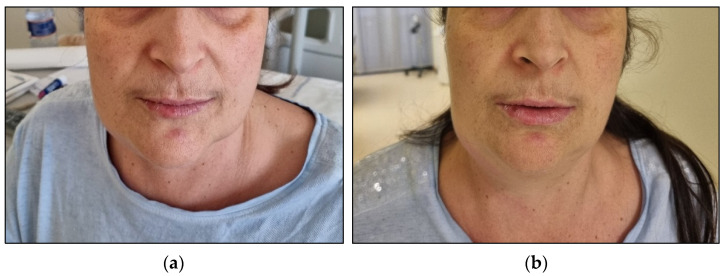
Differentiation syndrome. Submandibular swelling and lymphoadenopathy after 3 days of ATO+ATRA treatment (**a**) and after 6 days (**b**).

Since basophilic blasts are usually characterised by myeloid markers such as CD13 and CD33 and other markers such as CD123 and CD11b, the immunophenotype analysis concluded it was for basophilic leukosis. Considering the unusual results of cytogenetic evaluation and the basophilic phenotype, we ultimately decided to test the presence of PML::RARA rearrangement, which resulted positive for BCR3 transcript. Therefore, we confirmed the diagnosis of APL with basophilic differentiation. We treated the patient with ATO (10 mg/die) and ATRA (45 mg/m^2^/die), as it is the recommended treatment for standard risk APL. After three days of treatment, the patient presented bilateral swelling in the submandibular region, mainly on the left side, along with dysphonia and dysphagia. Suspecting a differentiation syndrome, we started treatment with metilprednisolone 1 mg/kg/die. In the following days, the patient experienced a further increase in the dimension of submandibular lymphadenopathies and developed an itchy skin rash with hives-like lesions (Figure 4).

Blood examination showed an increased leukocyte count (from 1400/mmc to 6020/mmc). Thus, therapy was modified, replacing metilprednisolone with dexamethasone 10 mg bid and suspending ATRA. Both rash and lymphadenopathies improved after a few days and the patient completed the induction treatment with ATO/ATRA without any further complications. She obtained a complete remission (CR) at bone marrow evaluation (less than 5% of blast cells in flow cytometry and undetectable PML::RARA) and partial haematologic recovery (Hb 9.9 g/dL, PLT 223.000/mcl, WBC 1260/mcl, ANC 830/mcl) at day +37 from the beginning of treatment. Currently, the patient has completed consolidation and maintains complete remission after a follow-up of 12 months.

## 4. Discussion

### 4.1. Cytogenic Alterations

Acute promyelocytic leukemia is characterised by the balanced translocation t(15;17) (q24;q21), found in almost 95% of cases. This translocation involves the promyelocytic leukemia (PML) gene on chromosome 15 and the retinoic acid receptor alpha (RARA) gene on chromosome 17, and results in the PML::RARA fusion gene [12]. The PML gene is located on chromosome band 15q24 and contains nine exons, which produce several alternatively spliced transcripts. All isoforms share the same N-terminal region (exons 1 to 3) but due to alternative splicing, differ in the central (exons 4, 5 and 6) or the C-terminal regions. PML transcripts interact with over 170 different proteins and are involved in the regulation of several cellular pathways affecting apoptosis, senescence, epigenetic regulation and transcription of haematopoietic stem cells. The RARA gene is located in chromosome band 17q21 and includes 10 exons encoding for two isoforms, RARA1 and RARA2, which differ from one another in the N-terminal Activation Function 1 (AF-1) domain. RARA is a nuclear receptor activated by retinoids and acts as a differentiating agent of myeloid haematopoietic cells through a ligand presence-dependent mechanism. In the presence of the ligands, RARA forms heterodimers with retinoid X receptor (RXR) cofactor, which then binds specific cis-acting motifs called retinoic acid responsive elements (RARE). In the absence of ligands, RARA/RXR dimers interact with nuclear corepressors, ultimately resulting in nucleosome assembly and transcriptional repression. The PML::RARA fusion protein has the ability to interact with several classes of protein, including the same PML and other transcription and epigenetic factors. This results in the repression of several genes involved in myeloid differentiation, producing a block of myeloid differentiation at the promyelocytic stage. On top of that, PML::RARA confers survival and proliferative advantage to leukemic cells, which leads to the progressive accumulation of promyelocytes in the bone marrow. It has been known since the early 1990s that the PML gene contains three breakpoint clusters, which results in different transcripts: the long bcr-1, the variant bcr-2 and the short bcr-3. Brc-1 and bcr-2 are the most common transcripts found in 95% of patients, while bcr-3 is rarer to detect, probably because it is the result of a more complex splicing [13,14].

The clinical and biological impacts of these transcript isoforms are still not fully assessed; the same studies were focused on the association of these isoforms with FLT3-ITD, a genetic alteration found in acute leukemia. FLT3-ITD is detected in 30–40% of APL patients and seems to be linked to higher WBC count and higher risk disease, but, to date, results are controversial. Apart from FLT3, there are some other recurrent genetic alterations that are also found in other acute myeloid leukemia subtypes, including WT1 (14%), NRAS (10%), KRAS (4%) and MYC amplification (12%). At the same time, other genetic alterations commonly found in acute myeloid leukemia, like DNMT3, TET2, NPM1, IDH1 or IDH2, are rarely detected, confirming the unique pathogenesis of APL [5]. 

Since the t(15;17) was described, other translocations involving the RARA gene have been found in acute leukemia patients presenting with typical clinical and/or morphological features of APL. Some of these rare cytogenetic alterations involving the RARA gene are listed in Table 1.

More rarely, fusions involving other retinoic acid receptors have also been detected [4].

### 4.2. t(12;17) Translocation in Acute Leukemia Has Been Described but t(12;15;17) Is Very Rare

As was already mentioned, a minority of patients with APL have either simple or complex translocations. Unfortunately, with conventional cytogenetic methods, these translocations can be misdiagnosed and PML::RARA can be masked with potentially severe results on treatment and prognosis. Our patient presented a variant three-way translocation which involved chromosome 12, 15 and 17 with breakpoints at 12p13, 15q24 and 17q21 and resulted in PML::RARA BCR3 transcript. We found only two cases reported in the literature of APL patients with complex translocations involving chromosomes 12, 15 and 17, but they were not associated with basophil differentiation [15,16].

A multi-step process was proposed to explain the pathogenetic mechanism of this three-way translocation. After a standard t(15;17) is formed, a translocation between chromosome 15 and chromosome 12 may occur and PML/RARA fusion genes move to chromosome 12. However, in our samples, we did not observe cells with only t(15;17) and this suggests that this process could also occur as a single event. 

Interestingly, there are some reports of cytogenetic aberrations involving chromosomes 12 and 17 in haematological malignancies. In particular, translocation t(12;17) (p13;q21) has been reported in 26 cases worldwide, mainly in patients with pre-B acute lymphoblastic leukemia (ALL), while only four cases of acute myeloid leukemia (AML) and a single case of mixed phenotype acute leukemia (MPAL) with t(12;17) (p13;q21) have been reported so far [17,18,19]. Notably, there is a relation between t(12;17) (p13;q21) rearrangement and specific antigens expression, since CD13 and/or CD33 are usually observed in reported cases [20,21].

### 4.3. Basophilic Differentiation in APL

Basophils are rare white blood cells in the bloodstream accounting for 0.01–0.3% of the leukocyte population. However, the small proportion is easily distinguished by large cytoplasmic granules, which are dark blue coloured reacting with specific dyes. Basophilia is commonly associated with chronic myeloid leukemia, in the accelerated phase or during blast crisis. It is also associated with other myeloproliferative neoplasms, but its association with acute leukemia is very rare and it has been described in association with acute basophilic leukemia [22]. There are some reports of basophilic differentiation of acute promyelocytic leukemia cells, which is associated with poor treatment response and high haemorrhagic risk [23,24]. The expression of the CD203c and/or CD22 basophil-associated markers showed the strongest association with the occurrence and severity of bleeding [6,25]. In contrast to what was previously reported in the literature and, despite presenting CD22 positivity in flow cytometry, our patient did not show any bleeding manifestations and obtained a complete response after treatment with ATO/ATRA. However, the patient experienced various symptoms caused by hyperhistaminemia. It is well known that in classic acute promyelocytic leukemia, ATRA induces differentiation of leukemic promyelocytes into mature neutrophils causing the so-called differentiation syndrome, which manifests with leukocytosis, weight increase, fever and ipotension. In basophilic APL, the leukemic promyelocytes probably differentiate into basophils, causing unusual differentiation syndrome symptoms due to the release of histamine, heparin and cytokines involved in anaphylaxis by basophils. We did not observe a basophils increase during the course of treatment, but our patient experienced various episodes of skin rash and itching, which were successfully treated with endovenous antihistamine therapy [26].

## 5. Conclusions

In the last twenty years, the history of acute promyelocytic leukemia has dramatically changed, from being a fatal disease to becoming one of the most curable forms of leukemia. However, in some cases, diagnosis may be challenging due to the atypical presentation of the disease. Although most patients carry the t(15;17) translocation, other cytogenetic abnormalities have been reported in the literature as causing the creation of the PML::RARA gene through other mechanisms. In this paper, we described a peculiar case of APL presenting with basophilia caused by a three-way translocation involving chromosomes 12, 17 and 15. Both the presence of an additional chromosome to the usual APL translocation and the basophilic differentiation did not prejudice the therapeutic outcome of conventional ATRA-ATO therapy and the patient managed to reach CR and, currently, still maintains CR after 12 months of follow-up. On the other side, basophilia may have contributed to the development of unusual differentiation syndrome, which resembled an allergic reaction and responded to antihistaminic and corticosteroids. According to this, steroids and anti-histamine therapy should be considered valid options in the treatment of differentiation syndrome in this peculiar set of patients. Lastly, bleeding events are frequently associated with basophilic differentiation in APL (especially with CD22 and CD203 immunophenotypes). However, our patient did not experience similar events.

## Figures and Tables

**Table 1:** APL cytogenetic alterations involving the RARA gene reported in [4,5].

Cytogenetic Abnormality	Fusion Gene	Incidence
t(15;17) (q22;q21)	PML::RARA	98%
t(11;17) (q23;q21)	ZBTB16::RARA	1%
t(X;17) (p11;q21)	BCoR::RARA	2 cases
t(X;17) (p28;q12)	X::RARA	1 case
t(4;17) (q12;q21)	FIP1L1::RARA	4 cases
t(3;17) (q26;q21)	TBLR1::RARA	4 cases
t(3;17) (q26;q21)	FNDC3B::RARA	1 case
t(7;17) (q11;q21)	6TF2I::RARA	1 case
t(1;17) (q42;q21)	IRF2BP2::RARA	6 cases
t(2;17) (q32;q21)	OBFC2A::RARA	1 case
t(5;17) (q35;q21)	NPM1::RARA	11 cases
t(11;17) (q13;q21)	NuMA::RARA	1 case
del(17) (q21q24)	PRKAR1A::RARA	1 case
t(17;17) (q21;q21)	STAT5b::RARA	17 cases
t(17;17) (q21;q21)	STAT3::RARA	2 cases
t(17;17) (q21;q24)	PRKAR1A::RARA	2 cases
t(14;17) (q11;q21)	HNRNPC::RARA	1 case
t(3;14;17) (q12;q11;q21)	TFG::RARA	1 case

## Data Availability

Data are contained within the article.
